# Effect of a novel somatostatin analogue combined with cytotoxic drugs on human tumour xenografts and metastasis of B16 melanoma

**DOI:** 10.1038/sj.bjc.6600668

**Published:** 2003-01-28

**Authors:** B Szende, A Horváth, G Bökönyi, G Kéri

**Affiliations:** 1 1st Department of Pathology and Experimental Cancer Research, Semmelweis University and Molecular Pathology Research Group Joint Research Organisation of the Hungarian Academy of Sciences, and Semmelweis University Budapest, Hungary; 2 Department of Medical Chemistry, Molecular Biology and Pathobiochemistry, Semmelweis University, Peptide Biochemistry Research Group Joint Research Organisation of the Hungarian Academy of Sciences and Semmelweis University Budapest, H-1085 Budapest, Üllõi u. 26, Hungary

**Keywords:** TT-232, somatostatin, Dacarbazine, Etoposide, melanoma, lymphoma

## Abstract

A novel somatostatin analogue, TT-232 (which inhibits the proliferation of various cell cultures and transplantable mouse tumours), was examined regarding its effect on human melanoma and lymphoma xenografts as a single treatment or in combination with DTIC (dacarbazine) and etoposide. TT-232 inhibited the growth of HT-18 melanoma xenografts, a dose of 5 mg kg^−1^ being the most effective. Combination of 1 mg kg^−1^ TT-232 with 30 or 60 mg kg^−1^ DTIC (administered daily) resulted in a stronger inhibitory effect compared to TT-232 or DTIC as a single modality. Antimetastatic effect of TT-232 treatment combined with DTIC was studied using the B16 mouse melanoma muscle – lung metastasis model. The number of lung metastases of B16 melanoma could be decreased by the daily administration of 1 mg kg^−1^ TT-232 or 60 mg kg^−1^, but not of 30 mg kg^−1^ DTIC. TT-232, combined with 30 or 60 mg kg^−1^ DTIC decreased the lung metastasis number significantly lower than the control. Nearly 50% growth inhibition of HT-58 lymphoma was achieved by daily treatment with 1 mg kg^−1^ TT-232. 5 mg kg^−1^ etoposide, administered daily, resulted in a similar effect. The combination of 1 mg kg^−1^ TT-232 and 5 mg kg^−1^ etoposide was significantly more effective than TT-232 or etoposide as a single treatment. The very strong tumour growth inhibitory effect of 10 mg kg^−1^ etoposide could even be increased by combination with TT-232. These experimental data suggest that TT-232 may be an effective new tool in the combination chemotherapy of malignant tumours like melanoma and lymphoma.

A novel somatostatin analogue of a five-residue ring structure D-Phe-Cys-Tyr-D-Trp-Lys-Cys-Thr-NH_2_ (TT-232) has been studied for its antiproliferative activity by our group. TT-232 showed a strong antitumour effect both *in vitro* and *in vivo*. TT-232 was effective on transplanted animal tumours (COLO 26, B_16_ melanoma, S180 sarcoma) and on human tumour xenografts (MDA-MB-231 human breast cancer, PC-3 human prostate cancer). It was a very important and promising finding that TT-232 proved to be very effective in the D-10 melanoma model (Kéri *et al*, 1996; Tejeda *et al*, 1999; Schwab *et al*, 2001). In addition, TT-232 had practically no growth hormone release inhibitory activity either in superfused rat pituitary cells or in rats *in vivo* (Kéri *et al*, 1993a, 1993b, 1996), while the application of various other somatostatin analogues (Robbins, 1996; Schally, 1988; Janecka *et al*, 2001) in tumour therapy is limited because of their endocrine side effects (Setyono-Han *et al*, 1987). TT-232 was shown to be a potent inducer of apoptosis in a wide array of cancer cell lines *in vitro* and in animal models *in vivo*. On the other hand, it does not appear to produce the endocrine effects of the natural compound (Kéri *et al*, 1996). The peptide acts via short-term induction of tyrosine phosphatases and inhibition of tyrosine kinases: these two effects are most probably independent of each other (Vántus *et al*, 1995, 2001). We demonstrated that early transient activation of Erk/MAPK is important for the induction of cell cycle arrest and that activation of the Erk/MAPK pathway by TT-232 involves PI 3-kinase, PKC*δ* and the protein tyrosine phosphatase *α* (PTP*α*) (Steták *et al*, 2001b). Moreover, we demonstrated interaction of PI 3-kinase and PKC*δ* with PTP*α* and showed that the tyrosine phosphatase plays a role in the activation of MAPK (Steták *et al*, 2001a). Intracellular target molecules, like the KU-86 protein, have been investigated in the TT-232 mechanism of action, but unlike for octreotide, intracellular distribution was not altered after treatment, suggesting a p53-independent apoptotic mechanism. (Tóvári *et al*, 1998). *In vivo* data obtained so far proved selectivity of TT-232 concerning its antitumour activity: there was no bone marrow suppression, or sign of any toxicity including weight loss of experimental animals even in doses exceeding the therapeutic dosage (Kéri *et al*, 1996; Tejeda *et al*, 1999, 2000; Schwab *et al*, 2001). These observations suggest that somatostatin analogues may be of value in the treatment of various human tumours, especially in combination with well established and already widely used antitumour drugs.

In order to study this therapeutic potential, two different tumour lines and two different anticancer drugs were used in our experiments to be presented. Since TT-232 proved to be effective against melanoma, HT-18 human melanoma xenograft was chosen as one of the experimental models. The known anticancer drug in this case was dacarbazine (DTIC) (Wagner *et al*, 2000). The other chemotherapeutic agent was etoposide, which has a different mechanism of action, that is, it induces apoptosis (Hauke and Armitage, 2000). Etoposide is generally used in the therapy of lymphomas, therefore a human lymphoma xenograft, HT-58, was used.

## Materials and methods

### Animals and animal-keeping conditions

Inbred, 8-week-old male CBA mice (Charles River Ltd, Hungary) were used in the experiments applying human tumour xenografts. Inbred, 8-week-old male C_57_Bl/6 mice (Charles River Ltd., Hungary) were used for inoculating B16 melanoma. The animals were housed in an airconditioned, specific pathogen-free Animal Care Facility in our department under controlled humidity (55±5%) and temperature (23±2°C). They were caged (5 per cage) in polyethylene boxes and had free access to CRLTN standard pelleted laboratory rodent chow (Charles River Ltd, Hungary) and tap water.

All animal experiments have been performed following the requirements (243/1998. XII. 31.) of the Hungarian Government, and after permission of the Animal Welfare Controlling Office, Budapest, Hungary (No 25-133/2001), meeting the standards required by the UKCCCR guidelines (Workman *et al*, 1998).

### Tumours

The human malignant melanoma HT-18 (ATCC CCL 121) cell line was obtained from NIH/NCI, Bethesda, MD, USA; the human B-cell lymphoma cell line HT-58 was established in our department (Kopper *et al*, 1991). All cell lines were maintained in culture at the Cell Culture Laboratory of our department. Cultured cells (2×10^6^) in log phase were inoculated subcutaneously into immunodeprived mice (see below) and the subcutaneously growing tumours were further transplanted three times in 3 week intervals. Experiments were started from the fourth passage of the tumours.

The transplantable mouse melanoma line B16 was obtained from the Institut für Tumorbiologie und Krebsforschung, Universität Wien, Austria (courtesy Dr W Bursch) and maintained by serial transplantation at our Animal Care Facility, by intramuscular injection of 10^5^ suspended tumour cells into the muscles of the right thigh.

### Preparation of immunodeprived mice and tumour inoculation

**Figure 1:**
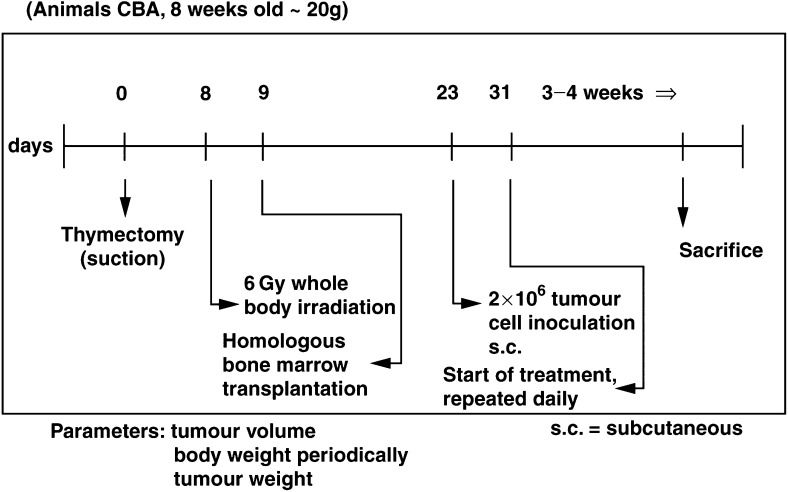
Experiment with human tumour xenografts on immunodeprived mice.

The 8-week-old male CBA mice were thymectomised by suction thymectomy. After 8 days, 6 Gy whole-body irradiation was given; followed within 24 h by homologous bone marrow transplantation. At day 23 after thymectomy, 2×10^6^ tumour cells were inoculated subcutaneously (Figure 1

).

### Compounds administered

TT-232 (D-Phe-Cys-Tyr-D-Trp-Lys-Cys-Thr-NH_2_) is the product of our Laboratory. DTIC (Dacarbazine), an alkylating agent with inhibitory effect on purine synthesis, was obtained from Lachema, Brno, Czech Republic. The ampoules contained 100 mg DTIC in powder form. Etoposide, a semisynthetic podophyllotoxine derivative, was purchased from EVA, Haarlem, The Netherlands. The ampoules contained 200 mg etoposide in 10 mg sterile parenteral solution.

### Treatment schedule

#### *Experiment 1* (HT-18 melanoma, TT-232 treatment)

Treatment with TT-232 (0.2 – 1.5 mg kg^−1^ body weight) was started on day 31 after thymectomy. TT-232 was dissolved in 0.9% sodium chloride and DMSO mixture (20 : 1) and applied daily subcutaneously in aliquots of 0.2 ml. Control mice received only the solvent in aliquots of 0.2 ml s.c.

#### *Experiment 2* (HT-18 melanoma, TT-232 and DTIC treatment)

Treatment with TT-232 (1 mg kg^−1^ body weight) was started on day 31 after thymectomy. Dissolution and application as in Experiment 1.

DTIC administration was started the same day as TT-232. DTIC was dissolved in 3% mannitol.

The daily dose of DTIC was 30 mg kg^−1^ body weight and 60 mg kg^−1^ body weight, respectively. DTIC was administered i.p. The following groups of 10 were studied: Control (treated i.p. with solvent only), TT-232 (1 mg kg^−1^), DTIC (30 mg kg^−1^), DTIC (60 mg kg^−1^), TT-232 (1 mg kg^−1^) + DTIC (30 mg kg^−1^), TT-232 (1 mg kg^−1^) + DTIC (60 mg kg^−1^).

### Follow-up and termination of the experiments with xenografts

Body weight was regularly determined at 4- to 6-day intervals throughout the experiment. The experiments were terminated on day 53 by exsanguination under Nembutal anaesthesia. Blood serum was preserved for other studies (not presented here). Body and tumour mass as well as tumour volume (measuring two diameters using a caliper square) was recorded in order to avoid occasional discrepancies between the values obtained by one of these methods. The tumour tissues of all animals were fixed for histological examination (to be reported).

Statistical analysis of the data on body mass, tumour volume and tumour mass was performed using Student's *t*-test. In all, 10 mice/group were used in all experiments for each dose and for the solvent-treated controls.

#### *Experiment 3* (B16 melanoma, TT-232 and DTIC treatment)

**Figure 2:**
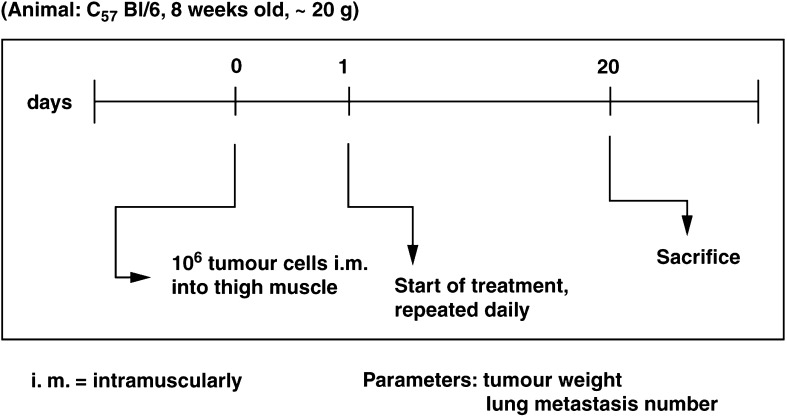
Experiment with B16 mouse melanoma.

The B16 melanoma cells were inoculated intramuscularly (see above). In summary, 24 h after tumour inoculation treatment was started and repeated daily until day 20 after tumour inoculation. At this point in time the experiment was terminated by exsanguination under Nembutal anaesthesia. Body masses and weight of the primary, intramuscularly growing tumour were measured. The lungs were fixed in Bouin's fixative and the number of lung metastases was counted under an inverted stereo microscope 2 days later (Figure 2

). The following doses, in aliquots of 0.2 ml, were used: TT-232, 1 mg kg^−1^ body weight subcutaneously; DTIC, 30 mg kg^−1^ body weight intraperitoneally; DTIC, 60 mg kg^−1^ body weight intraperitoneally. The following groups were formed: TT-232 (1 mg kg^−1^), DTIC (30 mg kg^−1^), DTIC (60 mg kg^−1^), TT-232 (1 mg kg^−1^) + DTIC (30 mg kg^−1^), TT-232 (1 mg kg^−1^) + DTIC (60 mg kg^−1^). Dissolution and application as in Experiments 1 and 2. Controls were treated subcutaneously, daily with 0.2 ml solvent (see above). In all, 10 mice were used for all doses and the control. Student's *t*-test was used for statistical analysis.

#### *Experiment 4* (HT-58 lymphoma, TT-232 and etoposide treatment)

Treatment with TT-232 (1 mg kg^−1^ body weight) was started on day 27 after thymectomy. Dissolution and application as in Experiment 1. Etoposide administration was started the same day as that of TT-232. Etoposide was dissolved in 0.9% sodium chloride. The dose of etoposide was 5 and 10 mg kg^−1^ body weight, respectively. Etoposide was administered daily i.v. The following groups were formed: control (treated subcutaneously with the solvent only), TT-232 (1 mg kg^−1^), etoposide (5 mg kg^−1^), etoposide (10 mg kg^−1^), TT-232 (1 mg kg^−1^) + etoposide (5 mg kg^−1^), TT-232 (1 mg kg^−1^) + etoposide (10 mg kg^−1^).

## Results

### *Experiment 1* (HT-18 melanoma, TT-232 treatment)

As shown in Table 1

**Table 1:** Effect of TT-232 treatment on tumour weight and tumour volume of HT-18 melanoma xenografts

	**Dose of TT-232**			
	**Control**	**0.2 mg kg^−1^**	**1.0 mg kg^−1^**	**5.0 mg kg^−1^**
Tumour weight (g) (mean±s.d.)	0.88±0.33	0.54±0.44	0.55±0.35	0.44±0.24*
Tumour volume at termination (mm^3^) (mean±s.d.)	217.45±38.51	199.09±82.67	171.00±97.8	138.42±59.99**

* *P*<0.003 *vs* control

** *P*<0.002 *vs* control.

, TT-232 inhibited the growth of HT-18 human melanoma xenografts, reflected in tumour volume and tumour weight at termination of the experiment. The strongest inhibition was observed after the administration of 5 mg kg^−1^ TT-232 (50%), but the two lower doses also retarded tumour growth, although not significantly (approximately to the same extent, 30%). Based on the data of our previous experiments (Kéri Gy *et al*, 1966; Tejeda *et al*, 1999; Schwab *et al*, 2001) the dose of 1 mg kg^−1^ was selected for experiments when TT-232 was applied in combination with other compounds.

### *Experiment 2* (HT-18 melanoma, TT-232 and DTIC treatment)

The results of these experiments are summarised in Table 2

**Table 2:** Effect of TT-232 and DTIC treatment on tumour weight and tumour volume of HT-18 melanoma xenografts

	**Tumourweight (g)**	**Tumour volume at termination (mm^3^)**
**Treatment**	**(mean±s.d.)**	**(mean±s.d.)**
Control	1.88±1.28	253.87±87.9
TT-232 1 mg kg^−1^	1.20±1.16	227±98.15
DTIC 30 mg kg^−1^	0.90±0.96	206.5±58.44
DTIC 60 mg kg^−1^	0.82±0.77	235.37±127.04
TT-232 1 mg kg^−1^+DTIC 30 mg kg^−1^	0.69±0.72*	207.14±123.28
TT-232 1 mg kg^−1^+DTIC 60 mg kg^−1^	0.68±0.46**	168.11±116.26

* *P*<0.02 *vs* control

** *P*<0.01 *vs* control.

. Interestingly, there was no difference between the effects of 30 and 60 mg kg^−1^ DTIC. A measure of 1 mg kg^−1^ TT-232 exerted an inhibitory effect in the same range observed in Experiment 1. The combination of TT-232 with DTIC resulted in a stronger effect compared to either TT-232 or DTIC alone. Both doses of DTIC, in combination with TT-232, resulted in an equal degree of tumour growth retardation (62 – 63%) showing significant difference from the control.

### *Experiment 3* (B16 melanoma, TT-232 and DTIC treatment)

The growth of the intramuscularly growing primary B16 melanoma could only be retarded to a certain degree in the groups treated with 30 or 60 mg kg^−1^ DTIC. No difference occurred between the effect of the two doses and the addition of TT-232 did not increase the effect of DTIC on the primary tumour. However, the number of lung metastases was markedly decreased in the TT-232-treated animals compared to the controls. This effect equalled that of 60 mg kg^−1^ DTIC, whereas 30 mg kg^−1^ DTIC was ineffective. When TT-232 was combined with 30 or 60 mg kg^−1^ DTIC, the metastasis number decreased to a value that was significantly lower compared to the controls (Table 3

**Table 3:** Effect of TT-232 and DTIC treatment on the primary tumour weight and lung metastasis number of B16 mouse melanoma

	**Tumour weight (g)**	**Metastasis number**
**Treatment**	**(mean±s.d.)**	**(mean±s.d.)**
Control	4.9±0.9	9.7±9.4
TT-232 1 mg kg^−1^	4.7±1.1	4.6±4.8
DTIC 30 mg kg^−1^	3.8±1.1*	7.0±7.1
DTIC 60 mg kg^−1^	3.6±0.7**	4.28±4.95
TT-232 1 mg kg^−1^+DTIC 30 mg kg^−1^	4.0±0.6*	2.16±1.32*****
T-232 1 mg kg^−1^+DTIC 60 mg kg^−1^	3.6±1.0****	2.2±1.7*****

* *P*<0.03 *vs* control

** *P*<0.002 *vs* control and 0.01 *vs* TT-232 1 mg kg^−1^

**** *P*<0.01 *vs* control

***** *P*<0.02 *vs* control.

).

### *Experiment 4* (HT-58 lymphoma, TT-232 and etoposide treatment)

The results shown in Table 4

**Table 4:** Effect of TT-232 and etoposide on the tumour weight and tumour volume of HT-58 lymphoma xenografts

	**Tumourweight (g)**	**Tumour volume at termination (mm^3^)**
**Treatment**	**(mean±s.d.)**	**(mean±s.d.)**
Control	0.73±0.33	194±142
TT-232 1 mg kg^−1^	0.38±0.14*	58±48*
Etoposide 5 mg kg^−1^	0.28±0.25**	56±33*
Etoposide 10 mg kg^−1^	0.05 ±0.03***	14±4**
TT-232 1 mg kg^−1^+etoposide 5 mg kg^−1^	0.17±0.03**	34±33**
TT-232 1 mg kg^−1^+etoposide 10 mg kg^−1^	0.02±0.0***	12±0***

* *P*<0.05 *vs* control

** *P*<0.01 *vs* control and 0.01 *vs* TT-232 1 mg kg^−1^

*** *P*<0.002 *vs* control.

point to the fact that nearly 50% tumour growth inhibition could be achieved by 1 mg kg^−1^ TT-232 treatment. A measure of 5 mg kg^−1^ etoposide resulted in a similar effect and the combination of TT-232 and 5 mg kg^−1^ etoposide was significantly more effective than TT-232 or etoposide as a single treatment. In total, 10 mg kg^−1^ etoposide alone inhibited tumour growth very strongly, but this effect could even be increased by the combination of 10 mg kg^−1^ etoposide with 1 mg kg^−1^ TT-232.

## Discussion

Our experiments showed that even TT-232 monotherapy inhibited significantly the growth of HT-18 melanoma. Combination of TT232 with DTIC resulted in a significant antiproliferative effect compared to the control. Similarly, the lung metastasis number of B16 melanoma could be decreased to a value significantly lower than the control by TT-232 combined with DTIC, in relatively low daily doses. Similar results were registered when HT-58 lymphoma-bearing mice were treated with TT-232 alone or in combination with etoposide. The 1 mg kg^−1^ dose of TT-232 proved to be less effective than 5 mg kg^−1^, but was selected for combination studies in order to find out whether the marginal effect exerted by this dose can be enhanced by combination with other compounds.

DTIC is an alkylating agent that inhibits purine synthesis (Wagner *et al*, 2000). Etoposide, a semisynthetic derivative of podophyllotoxine is an inhibitor of DNA synthesis and acts in the S and G_1_ phase of the cell cycle (Moskowity *et al*, 1999; Mugitani *et al*, 1999). Both compounds are used in the therapy of human lymphomas, but DTIC is primarily applied against malignant melanomas. In our experiments, particularly in Experiment 2, the tumour growth inhibitory effect of DTIC was moderate. The lack of difference between the effect of 30 and 60 mg of DTIC in Experiment 2 may be explained by the possible suppressing effect of the higher dose on the cellular immune response of the mice thereby preventing cytotoxic lymphocytes to support the effect of the cytotoxic drug.

Our group and others have recently studied the mode of action of TT-232. The somatostatin analogue, TT-232, inhibits cell proliferation and induces apoptosis in a variety of tumour cells both *in vivo* and *in vitro*. As a result of the short half-life of the natural somatostatin, analogues were designed to enable clinical application. Introducing amino acids with ‘D’ configuration resulted in compounds much more resistant to tissue proteases, the major source of elimination of the natural peptide (Patel *et al*, 1996). This is characteristic for the structure of all commercially available analogues and TT-232 as well. A number of these drugs, in turn, proved selectivity for different receptor subtypes of the somatostatin receptor family, which was associated with a marked difference in the 3D structure, while TT-232 had a unique 3D structure as was demonstrated by NMR studies (Kéri *et al*, 1993b; Jaspers *et al*, 1994).

We demonstrated the binding of the somatostatin analogue TT-232 to plasma membranes either *in vitro* (Szegedi *et al*, 1999) or *in vivo* with [^3^H]-TT-232. Moreover, selective binding of TT-232 to somatostatin receptor subtypes 1 (IC_50_ 713 nM) and 5 (IC_50_ 564 nM) has also been shown *in vitro* (Steták *et al*, 2001b) suggesting a receptor-mediated signalling mechanism. This finding is supported by the facts that TT-232 is a structural analogue of somatostatin, that the early signalling pathway of TT-232 is pertussis toxin sensitive, and several signalling elements are common between TT-232 and somatostatin. However, longer treatment by the compound leads to strong induction of apoptosis, which appears to be independent of early signaling events, and requires endocytosis (Vántus *et al*, 2001). This suggests a dual effect of TT-232 on cells, including an early signalling cascade via somatostatin receptors, and a second, later event through a so far unidentified pathway.

One of the early effects of the somatostatin analogue TT-232 is the rapid activation of protein tyrosine phosphatases and the induction of cell cycle arrest via the increase of p21^Cip1/Waf1^ levels (Steták *et al*, 2001b). This antiproliferative activity is mediated through a signaling pathway involving PI 3-kinase, pp60^c-src^ kinase and MAP-kinase (Steták *et al*, 2001a).

Combination of anticancer compounds with different modes of action is widely used in the therapy of malignancies. Since the chemotherapy of malignant melanoma is still unsolved (Van der Esch *et al*, 1981; Ak *et al*, 2000) and the situation is similar in the case of most lymphomas (Hauke and Armitage, 2000; Kluin-Nelemans *et al*, 2001), new therapeutic modalities are needed. Our experiments indicate that TT-232 could be successfully used in combination chemotherapy of melanomas and lymphomas, and probably also of other malignant tumours.
